# New Insight into Plant Saline-Alkali Tolerance Mechanisms and Application to Breeding

**DOI:** 10.3390/ijms232416048

**Published:** 2022-12-16

**Authors:** Yibo Cao, Huifang Song, Lingyun Zhang

**Affiliations:** Research & Development Center of Blueberry, Key Laboratory of Forest Silviculture and Conservation of the Ministry of Education, Beijing Forestry University, Beijing 100083, China

**Keywords:** saline-alkali stress, osmotic stress, Na^+^ toxicity, pH stress, HCO_3_^−^/CO_3_^2−^ stress

## Abstract

Saline-alkali stress is a widespread adversity that severely affects plant growth and productivity. Saline-alkaline soils are characterized by high salt content and high pH values, which simultaneously cause combined damage from osmotic stress, ionic toxicity, high pH and HCO_3_^−^/CO_3_^2−^ stress. In recent years, many determinants of salt tolerance have been identified and their regulatory mechanisms are fairly well understood. However, the mechanism by which plants respond to comprehensive saline-alkali stress remains largely unknown. This review summarizes recent advances in the physiological, biochemical and molecular mechanisms of plants tolerance to salinity or salt- alkali stress. Focused on the progress made in elucidating the regulation mechanisms adopted by plants in response to saline-alkali stress and present some new views on the understanding of plants in the face of comprehensive stress. Plants generally promote saline-alkali tolerance by maintaining pH and Na^+^ homeostasis, while the plants responding to HCO_3_^−^/CO_3_^2−^ stress are not exactly the same as high pH stress. We proposed that pH-tolerant or sensitive plants have evolved distinct mechanisms to adapt to saline-alkaline stress. Finally, we highlight the areas that require further research to reveal the new components of saline-alkali tolerance in plants and present the current and potential application of key determinants in breed improvement and molecular breeding.

## 1. Introduction

Saline-alkali stress is one of the major adverse factors affecting plant growth and yield formation. More than 1 billion hectares of the world’s land are disturbed by soil salinization and this problem continues to deteriorate with global climate change and unreasonable irrigation practices [1,2,3]. Saline-alkaline soils are usually characterized by high salt and high pH (above pH 8.0). In the natural environment, salt stress is mainly caused by neutral salts (e.g., NaCl, Na_2_SO_4_), whereas saline-alkali stress is usually induced by basic salts (e.g., NaHCO_3_, Na_2_CO_3_) [3,4]. Existing knowledge has shown that salt stress limits plant growth in two ways: during the initial phase, the water potential in the soil around the root system rapidly decreases, which results in further osmotic stress and the difficulty of root water absorption, severely inhibiting new leaf growth; then the high concentrations of salt causes an excessive accumulation of Na^+^ in plants that leads to ion toxicity [2], which is mainly manifested in accelerating the senescence of the leaves [5]. Most of the cereals (e.g., rice, maize and wheat) are sensitive to salt stress, salinity inhibiting the roots to absorb nutrients, reducing carbon fixation and photosynthesis, and eventually causing yield reduction [6,7]. For woody plants such as fruit trees, most of which are grown in poor site conditions and are more susceptible to salt stress. In apple, soil salinity affects the root architecture of apple rootstocks and greatly restricts the cultivation and yield of apples [8].

**Figure 1:**
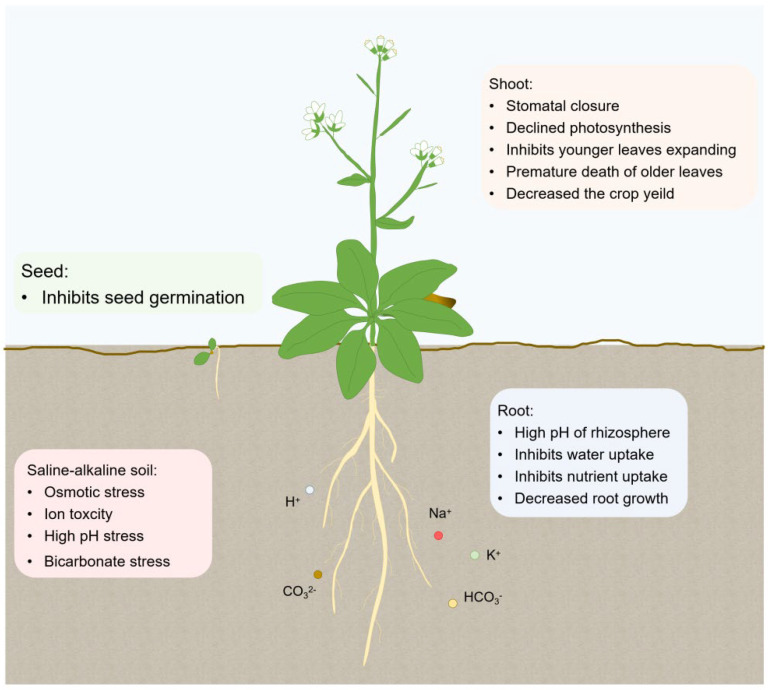
The effects of saline-alkali stress on the growth and development of plants. Saline-alkali stress induced osmotic stress, ion toxicity, high pH stress, and HCO_3_^−^/CO_3_^2−^ stress, which affect seed germination, disturb the pH of the rhizosphere, inhibit the absorption of water and nutrients in the root, leading to the decreased root growth. Meanwhile, osmotic stress affects stomatal closure, inhibits the expansion of younger leaves and accelerates the senescence of leaves, ultimately resulting in repressed photosynthesis and low crop yield.

Under normal conditions, the pH around the rhizosphere of plants is lower than the cytosolic pH of root cells (approximately 7.0), thus to form the transmembrane proton gradient driving nutrients absorption [9,10,11]. Saline-alkali stress increases the pH of the plant rhizosphere compared with neutral salt, which disrupts the proton gradient across the plasma membrane, and affects the physiological processes such as nutrient absorption and ion transport, ultimately leading to the physiological metabolism disorders that limit plant growth and even death [12,13]. In a word, saline-alkaline soil not only causes osmotic stress, ion toxicity, but also leads to high pH stress, oxidative stress, and bicarbonate/carbonate (HCO_3_^−^/CO_3_^2−^) stress for plants. In order to adapt to saline-alkaline stress, plants evolved to multiple strategies such as producing organic osmoticums, excluding excessive ion (e.g., Na^+^ and Cl^−^), acidifying the rhizosphere and scavenging ROS to alleviate saline-alkaline stress induced damage [14,15] (Figure 1).

Extensive studies on various plant species over the past few decades have contributed to elucidating the mechanisms by which plants respond to salt stress, and many genes involved in salt resistance have been identified. Generally, plant accumulated osmolytes (e.g., proline, glycinebetaine) to cope with osmotic stress under salt stress [16], and to circumvent the Na^+^ toxicity through Na^+^-preferring transporters (e.g., SOS1 and HKT1) [17,18,19]. Several studies have also shown that plants grown in saline-alkaline soil can reduce the pH of the rhizosphere to resist high pH stress by PM H^+^-ATPase-mediated root-to-soil H^+^ efflux [20]. However, studies focusing on salt in combination with alkali stress are still very scarce. To date, only a few potential genes were identified as involved in the response to saline-alkali stress (Table 1), and the mechanisms of plant adaptation to saline-alkali stress remain largely unknown.

In view of the universal distribution of combined saline-alkali stress in the world and the damage it causes to agricultural production, it is necessary to elucidate the molecular mechanism by which plants perceive and respond to salt or saline-alkali stress and to advance our understanding of the cultivation of saline-alkali tolerant crops through genetic engineering, and ultimately to promote the sustainable development of agriculture.

The following sections will focus on recent advances in plant tolerance to salt-alkali stress, including how plants perceive salt-alkali stress and the molecular mechanisms of plants response to salt-alkali stress. Finally, we highlight areas of research that needed further investigation to reveal the novel mechanisms of plants in response to saline-alkali stress.

## 2. Perceiving the Saline-Alkali Stress

Under saline-alkali conditions, plants perceive the changes in osmotic potential, cytosolic Na^+^ concentration and pH values and then activate the downstream signaling pathways to effectively respond to saline-alkali stress. In recent years, some signal molecules or proteins have been identified to be involved in the sensing of saline-alkali stress. Previous studies have shown that plants can perceive and respond to saline-alkali stress probably by regulating the change in Ca^2+^ concentration in different cell types. The increase of cytosolic Ca^2+^ induced by salt mainly occurs in the cortex and epidermal cells, whereas the rapid rise of cytosolic Ca^2+^ concentration induced by mannitol concentrates in epidermal cells [21]. The increases in cytosolic Ca^2+^ concentration was associated with the activation of Ca^2+^ channels located in the plasma membrane [22]. The plasma membrane calcium-permeable channel reduced hyperosmolality induced [Ca^2+^] increase 1 (OSCA1) is a putative sensor for hyper-osmotic stress and the calcium spike in the loss of function mutant for this gene was reduced under sorbitol and mannitol treatment [23]. In addition, the Ca^2+^-responsive phospholipid-binding proteins BONZAI (BON) are critical upstream regulators of plants response to early osmotic stress, which suppress nucleotide-binding leucine-rich repeat (NLR) signaling to release its inhibition of osmotic stress responses, thus improve plants osmotic stress tolerance [24]. The studies in Arabidopsis showed that glycosyl inositol phosphorylceramide (GIPC) located on the plasma membrane is the putative sensor for plants to perceive the changes in external Na^+^ concentration. *MOCA1* encodes the glucuronosyltransferase and is involved in the biosynthesis of GIPC. Under salt stress, Na^+^ binds to GIPCs to gate Ca^2+^ influx channels, promoting plant tolerance to salt stress [25]. High pH stress can also induce an increase in cytosolic Ca^2+^, which combine with the Ca^2+^ sensor (e.g., CBL7) to trigger the calcium signal pathway, and increase PM H^+^-ATPase activity by a complex interaction with HA family protein (e.g., AHA2 in Arabidopsis) [26]. A very recent important study report that plant cell-surface peptide-receptor complexes (RGF1-RGFRs and Pep1-PEPRs) can function as extracellular pH sensors. High pH leads to extracellular alkalinization, which inhibits the acidic-dependent interaction between root meristem growth factor 1 (RGF1) and its receptors (RGFRs) through sulfotyrosine, promoting the alkaline-dependent binding of plant elicitor peptides (Peps) to its receptors (PEPRs) through Glu/Asp [27]. However, the knowledge of how plants sense saline-alkali stress is very limited, and the definite HCO_3_^−^/CO_3_^2−^ receptors have not been identified. It is also not well understood how plants perceive the excess alkaline salts in the soil, whether other signaling molecules are involved in addition to Ca^2+^ signaling, and how the two types of stress signaling cross when plants are exposed to saline-alkali stress.

## 3. The Survival Strategies Adopted by Plants under Saline-Alkali Stress

### 3.1. Accumulation of Osmoregulatory Substances

Osmotic stress usually occurs in early stages of saline-alkali stress. Previous studies have shown that 40 mM NaCl can generate approximately 0.2 MPa osmotic potential, which would rapidly reduce the cell turgor pressure, shrink the plasma membrane, and ultimately inhibit plant growth [2]. Both salt and saline-alkali stress induce the accumulation of osmoregulatory substances (e.g., proline, betaine, sugars and polyamine) in plants [28,29,30]. Under osmotic stress, plants are able to accumulate more solutes to balance the extracellular osmolality, thus to maintain plants’ water content and to alleviate osmotic stress [31].

Proline is one of the free amino acids, and to date, many genes that regulate the synthesis and accumulation of proline have been identified in a variety of plant species. In Arabidopsis, lacking of δ-1-pyrroline-5-carboxylate synthetase (P5CS1) decreased proline synthesis, resulting in salt hypersensitivity [32,33]. Some transcription factors were also identified to participate in the accumulation of proline. Overexpressing *ZmDi19-1* from maize in Arabidopsis increased proline content, and the transgenic plants showed more tolerance to salt stress [34]. The transgenic Arabidopsis with *VaERF3* overexpression from adzuki bean accumulated more proline, while the content of malondialdehyde (MDA) and reactive oxygen species (ROS) were decreased [35]. Proline plays essential roles in salt and salt-alkali induced osmotic stress, however, the knowledge on the role of key genes and their regulatory mechanism in proline synthesis and accumulation is still limited.

With the widespread application of metabolomics, more and more osmoregulatory substances and key genes have been identified. Glycinebetaine is another important organic osmoticum that participate in the maintenance of plasma membrane structure integrity and enzyme activity under osmotic stress [36]. Sugars also act as an important osmotic adjustment in response to salt stress. Sucrose directly mediates the accumulation of D-phenylalanine, tryptophan and alkaloid to maintain osmotic potential in *Malus halliana* [37]. In apple, the S-acyltransferases MdPAT16 promoted sugar accumulation and thus enhanced plant tolerance to salt-induced osmotic stress [38]. Based on metabolic genome-wide analysis (mGWAS) analysis, 10 candidate genes in maize were identified to be significantly associated with the salt-induced osmotic stress (SIOS) tolerance and metabolite (METO) abundances. The phenotype indicated that a citrate synthase, a glucosyltransferase, and a cytochrome P450 were associated with the genotype of METO [16].

### 3.2. Ion Transporters Maintain Low Na^+^/K^+^ Ratio

Na^+^ is the most abundant soluble substance in saline or saline-alkaline soils. Under high external Na^+^ conditions, Na^+^ passively enters into the root cell via ion channels and transporters, such as non-selective cation channels (NSCCs) and K^+^ transporters (OsHKT2;1) [39,40]. Then the excessive Na^+^ is transported from root to shoot by transpiration, and thus leads to Na^+^ toxicity in plants [28]. Therefore, it is essential for plant survival to maintain Na^+^ homeostasis under saline-alkali stress. Previous evidence demonstrated that plants mainly adopt two strategies to circumvent Na^+^ toxicity, that is, maintaining Na^+^ homeostasis in the cytoplasm or reducing Na^+^ transport from root to shoot [2,28,41].

Plant reduction of Na^+^ accumulation in cytoplasm relies mainly on two ways: promoting Na^+^ efflux from the cell to the soil and compartmentalizing excessive Na^+^ into the vacuole [42]. Salt overly sensitive (SOS) signaling pathway was demonstrated to be mainly responsible for exporting Na^+^ out of the cell, which composed of SOS1 (a plasma membrane-located Na^+^/H^+^ antiporter), SOS2 (a CIPK family protein kinase) and SOS3 (a CBL family Ca^2+^-binding protein) [19]. Salt stress induces an instantaneous increase of cytosolic Ca^2+^ in the cell, and the Ca^2+^ signal was sensed and decoded by Ca^2+^-binding proteins SOS3 and SCaBP8/CBL10. The Ca^2+^ signal further promotes the interaction of SOS3 with SOS2 to form a complex, and then the activated SOS2 phosphorylates the Na^+^/H^+^ antiporter SOS1, thus promoting Na^+^ efflux and maintaining Na^+^ homeostasis in the cytoplasm [43,44,45,46]. The relatively conservative role of the SOS pathway in salt tolerance has been demonstrated in crops such as maize and tomato [47,48]. ZmSOS1 functions in root-to-rhizosphere Na^+^ efflux, SOS3/SOS2 complex (ZmCBL4 and ZmCBL8/ZmCIPK24a) phosphorylates ZmSOS1, thus activating its Na^+^-transporting activity in maize [47]. It has also been shown that small molecular compounds (SMC) play an important role in Na^+^ exclusion and plant salt tolerance by regulating the activity of SOS1. For instance, PLDα1-derived phosphatidic acid (PA) binds and activates MPK6, MKK7 and MKK9 to form MKK7/MKK9-MPK6 cascade, which can phosphorylate SOS1 to promote Na^+^ exclusion from plant cells [49,50]. Overexpression of *IbPSS1* (phosphatidylserine synthase) in sweet potato promoted the accumulation of phosphatidylserine (PS), activated plasma membrane Na^+^/H^+^ antiporter, thus to maintain cellular homeostasis and improve plant salt tolerance [51].

**Figure 2:**
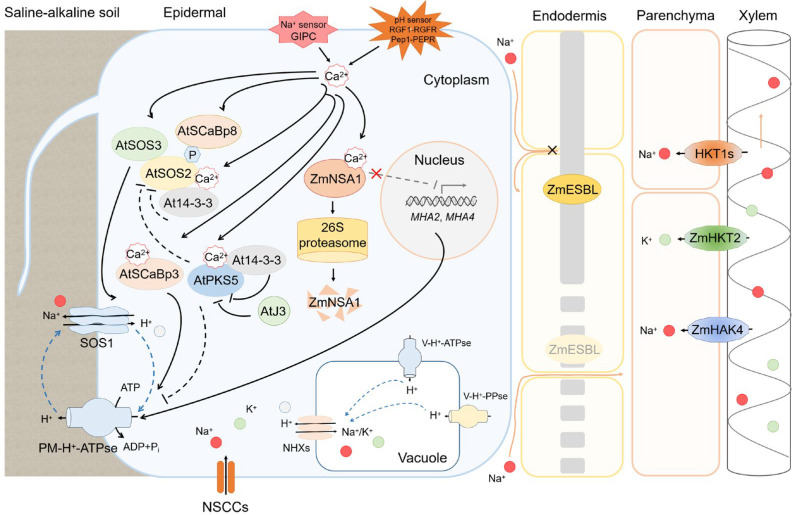
The mechanism model of ion (Na^+^ and K^+^) homeostasis and pH stability mediated by the combination of ion transporters and H^+^-ATPase. Na^+^ enters into the root cells via NSCCs. The change in Na^+^ and pH values was sensed by Na^+^ sensor GIPC or pH sensor and induced the change of cytoplastic Ca^2+^, which then binds to Ca^2+^-binding protein (SOS3, SCaBP8, SCaBP3 and ZmNSA1), triggers SOS pathway to promote Na^+^ exclusion. At the same time, J3 was activated by PKS5 to release the inhibition of AHA2, inducing SCaBP3 dissociation from AHA2, thus promoting PM H^+^-ATPase activity. In addition, Ca^2+^ binds to ZmNSA1 and triggers its degradation by the 26S proteasome, which induces the expression of *MHAs* and increases the activity of PM H^+^-ATPase. These pathways collectively mediate Na^+^ efflux under saline-alkali stress. NHXs, located in tonoplast, transport Na^+^ or K^+^ into vacuole dependent on the H^+^ gradient created by V-H^+^-ATPase and V-H^+^-PPase, and regulate the excessive Na^+^ compartmentalization, Na^+^/K^+^ balance and pH stability in cytoplasm. ZmESBL, located in Casparian strips, functions in the formation of CS and prevents excessive Na^+^ across the endodermis. The Na^+^-selective transporter HKT1 and ZmHAK4 mediate shoot Na^+^ exclusion by retrieving Na^+^ from the xylem vessels, while K^+^-preferring transporter HKT2 transports K^+^ from xylem sap to parenchyma cells, maintaining the lower Na^+^/K^+^ ratio under saline-alkali stress. NSCCs: non-selective cation channels.

The activity of SOS1 was dependent on the H^+^ gradient across the plasma membrane, which was established by the plasma membrane H^+^-ATPase (PM H^+^-ATPase) [19,52]. In Arabidopsis, *AHA2* encodes the PM H^+^-ATPase, which has an inhibitory region at the C-terminal, to which 14-3-3 protein can bind and activate AHA2, while the protein kinase PKS5 negatively regulates the activity of AHA2 [20]. Recent studies have also shown that the 14-3-3 protein acts as a Ca^2+^-dependent molecular switch mediating the activities of PKS5 and SOS2 to alleviate salt-induced Na^+^ toxicity (Figure 2) [53]. Existing studies have revealed that Na^+^ efflux mediated by the SOS pathway is associated with the activity of PM H^+^-ATPase and its regulators. The endogenous small molecules phosphatidylinositol (PI) was partially converted into 4-phosphate phosphatidylinositol (PI4P) to release PM H^+^-ATPase activity, thus activating SOS1 to increase plant salt tolerance [54]. PLDα (phospholipase D, phospholipids to produce PA) regulates the transcription level and activity of PM H^+^-ATPase to improve rice salt tolerance [55]. In sweet potato, the salt-tolerant cultivar accumulated more PS, thus to increase the activity of PM H^+^-ATPase and reduce K^+^-efflux, and delay salt-induced leaf senescence [56]. The endogenous free unsaturated fatty acids oleic acid (C18:1), linoleic acid (C18:2), and linolenic acid (C18:3) directly bound to the C-terminus of PM H^+^-ATPase AHA2 to activate its activity and promote plant salt tolerance [57]. These results suggest that small molecular compounds play a role in mediating plant salt tolerance by regulating PM H^+^-ATPase activity and more attention should be paid to excavate more SMC and uncover the underlying mechanism involved.

Compared with salt stress induced by neutral salt (NaCl), saline-alkali (NaHCO_3_) treatment aggravated the Na^+^ toxicity of plants [4,58], and our recent study in maize reports a QTL gene, *ZmNSA1* (*Na^+^ Content under Saline-Alkaline Condition*), encoding a Ca^2+^ binding protein that confers shoot Na^+^ variation and saline-alkali tolerance in maize [58]. Under saline-alkali conditions, the increased cytosolic Ca^2+^ binds to ZmNSA1 and triggers its degradation via the 26S proteasome, indirectly upregulating the expression level of *MHA2* and *MHA4*, which encode PM H^+^-ATPase, thereby enhancing the activity of PM H^+^-ATPase, promoting ZmSOS1-mediated root Na^+^ efflux and maize salt tolerance (Figure 2) [58]. As a result, saline-alkali stress induced high pH stress weakens the activity of ZmSOS1 and then aggravates Na^+^ toxicity in maize. Since the H^+^ gradient across the plasma membrane generated by PM H^+^-ATPase not only provides energy for Na^+^ transport, but also for the active transport of almost all nutrients [19,59], the key genes and proteins responsible for other anions (e.g., HCO_3_^−^, CO_3_^2−^ and PO_4_^3−^) or cations (e.g., K^+^, Mg^2+^) transport still need to be identified.

Compartmentalization of excessive Na^+^ into vacuole is another Na^+^ detoxification strategy for plants to cope with salt stress. Cation/H^+^ (NHX-type) antiporters are important regulators of intracellular ion homeostasis. NHX proteins were originally reported as Na^+^/H^+^ antiporters located in the tonoplast, and the transport activity of NHXs is dependent on the H^+^ gradient maintained by H^+^-ATPase and H^+^-PPase located in the tonoplast, SCaBP interacted with SOS2 to activate NHXs, leading to the sequestration of Na^+^ into the vacuole and conferring plant salt tolerance (Figure 2) [60,61,62,63]. However, existing studies showed that there was no direct correlation between increased salt tolerance and increased Na^+^ accumulation in the vacuoles by NHX proteins. In Arabidopsis, the double mutant *nhx1 nhx2* showed greater Na^+^ sequestration rates and similar salt tolerance compared to the wild type, but hypersensitive to external K^+^ [64,65]. The lack of any vacuolar NHX activity (*nhx1-4*) resulted in no K^+^ uptake, highly acidic vacuoles, and reduced but not abolished vacuolar Na^+^ uptake [66]. These studies suggest that NHXs mainly contribute to regulating vacuolar K^+^ and pH, and there is likely a cation/H^+^-independent Na^+^ conductive pathway in vacuoles. Despite investigations of the role of NHX proteins in promoting salt tolerance in plants, the mechanisms by which these transporters respond to saline-alkali stress are not well understood. The specific role of NHXs in Na^+^ entry into vacuoles and the key regulation factors of cation /H^+^-independent Na^+^ conductive pathway in vacuoles need to be further explored.

Na^+^ generally loads into stele through the apoplastic or symplastic pathway [2,67]. Casparian strip (CS) is lignin-based cell-wall modifications in the root endodermis of vascular plants, which act as an important barrier blocking the apoplastic transport of solutes and water [68]. Dirigent (DIR) family proteins have been reported to functions in guiding monolignols bond to formation of dimeric lignin [69]. Our recent study in maize revealed that a DIR protein ZmESBL mediated CS formation and confers to transpiration-dependent salt tolerance (TDST) in maize. ZmESBL plays a crucial role in the formation of the CS barrier, which prevent excessive Na^+^ across the endodermis to reach the stele through the apoplastic pathway and then blocked Na^+^ transport from the root to the shoot via xylem-based transpiration flow, thereby promoting shoot Na^+^ exclusion and salt tolerance. Furthermore, the orthologs of ZmESBL in Arabidopsis exhibit similar functions (Figure 2) [70]. In addition, the function of CS contributing to plants salt tolerance has also been reported in rice [71]. These studies demonstrated a conserved mechanism of lignin deposition in the endodermal CS domain of monocot and dicot species. As an important apoplastic transport barrier to prevent excessive ion transport across the endodermis, however, it remains to be further investigated whether saline-alkali stress induces CS reprogramming and to explore its function in maintaining nutrient uptake.

High affinity K^+^ transporter (HKT) family proteins are another ion transporters which function in regulating Na^+^ retrieval from the transpiration stream to reduce Na^+^ accumulation and maintain K^+^/Na^+^ balance in the shoot [40,72,73]. According to ion transport selectivity, the HKT family was divided into two subfamilies: class I functions as a Na^+^-preferring transporter, while class II shows K^+^ or Na^+^/K^+^ symport activities [40]. In Arabidopsis, the mutant of *AtHKT1;1* shows hypersensitivity to salt stress and accumulates more Na^+^ in shoots but less in roots; the concentration of Na^+^ in the xylem sap of *athkt;1* is also higher than in wild type, indicating the function of AtHKT1;1 in retrieving Na^+^ from the xylem sap and promoting leaf Na^+^ exclusion, thus enhancing plant salt tolerance [18]. In recent years, HKT1 has been identified in a range of plant species in combination with genome-wide analysis (GWAS) and quantitative trait loci (QTL) analysis, such as OsHKT1;5 in rice, TaHKT1;4 and TaHKT1;5 in wheat, HvHKT1;5 in barley, ZmHKT1 in maize and HKT1;1 in grapevine [72,74,75,76,77,78], etc. These studies showed that the functions and mechanisms of HKT1 family are similar in most of the identified plants, suggesting that the function of HKT1 family were conserved during the evolution of plant adaptation to salt stress. *HKT1* is specifically expressed in the pericycle and xylem parenchyma cells and functions as a conserved Na^+^-preferring transporter to withdraw Na^+^ from the xylem sap and prevent Na^+^ transport from root to shoot (Figure 2). Recent studies indicated that SET DOMAIN GROUP 721 (OsSDG721) protein positively regulates saline-alkali tolerance in rice by epigenetically regulating *OsHKT1;5* expression under saline-alkaline conditions [79,80]. Moreover, a Calcium/calmodulin-dependent protein kinase, OsDMI3, has been identified to improve saline-alkali tolerance by regulating Na^+^ and H^+^ influx in rice roots [81]. These findings demonstrated that HKT1-mediated Na^+^ homeostasis and its regulators also play crucial roles in plants saline-alkali tolerance, and further provide important genetic targets for breeding saline-alkaline-tolerant varieties.

Class II HKT functions as a crucial factor in regulating K^+^/Na^+^ ratio in plants. OsHKT2;1 mediates nutritional Na^+^ uptake under K^+^ starved conditions, and OsHKT2;4 shows K^+^, Ca^2+^ and Mg^2+^ permeability, both of which jointly function in maintaining K^+^ balance in plants [39,82,83]. *ZmHKT2* encodes a K^+^-preferring transporter that transports K^+^ from xylem sap to parenchyma cells, and the mutant maintains higher K^+^/Na^+^ ratio in shoot tissues to promote salt tolerance in maize (Figure 2) [73]. However, under the saline-alkali stress, whether the induced high pH change the Na^+^ transport activity of HKT1? Since saline-alkali stress caused the difficulty of nutrients uptake in plants, HKT family proteins probably function in transporting various cations (e.g., Na^+^, K^+^, Ca^2+^ and Mg^2+^) and what are the specific role of HKT2 in nutrients uptake under saline-alkali conditions?

In addition, high affinity K^+^ transporter (HAK) family protein also plays an important role in regulating K^+^/Na^+^ homeostasis under salt stress by selectively transporting Na^+^ or K^+^. In rice, the K^+^ transporter OsHAK1, OsHAK5, OsHAK16, and OsHAK21 promote rice tolerance to salt stress by regulating K^+^ uptake [84,85,86]. The most recent study also reported an endoplasmic reticulum-localized cytochrome b5 (OsCYB5-2), which can interact with OsHAK21 to promote OsHAK21-mediated K^+^-uptake and maintain intracellular K^+^/Na^+^ homeostasis [87]. OsHAK12 is a Na^+^-permeable transporter that mediates Na^+^ exclusion by retrieving Na^+^ from xylem vessels, thereby reducing Na^+^ accumulation and promoting salt tolerance [88]. Interestingly, ZmHAK4 is also a Na^+^-preferring transporter identified in maize and functions at Na^+^ concentration ranging from sub-millimolar levels to over 100 mM, unlike ZmHKT1 and ZmHKT1;2, which are reported to only function at high Na^+^ concentration (Figure 2) [89,90], suggesting that plants adopt different Na^+^ transport strategies under high or low Na^+^ conditions. In tomato, SlHAK20 transports Na^+^ and K^+^ to regulate K^+^/Na^+^ homeostasis under salt stress. Lack of SIHAK20 leads to plant hypersensitivity to salt stress and knockout of the rice homologous OsHAK4/OsHAK17 also lead to the same phenotype [91], suggesting the wide role of HAK family proteins in plant response to salt stress. Since HAK family has a large member in various plants (e.g., 13 members in Arabidopsis, 27 members in maize) [89,92], only a few member functions have been identified so far. There is no doubt that these ion transporters play a crucial role in maintaining the low Na^+^/K^+^ ratio in saline or saline-alkaline soil. However, the regulatory mechanisms of these transporters remain largely unknown. For example, the upstream regulator factors of *HKTs* or *HAKs,* or the other coupling signal pathway (e.g., SOS pathway) to maintain Na^+^ homeostasis need to be further identified.

### 3.3. Rhizosphere Acidification by H^+^-ATPase, Organic Acid and Rhizobacteria

High pH is another notable feature of saline-alkaline soil. Saline-alkali stress increased the pH value of the rhizosphere and restricted nutrients uptake, thus inhibiting plant growth [9,93]. It has been shown that plants rely on PM H^+^-ATPase to export proton (H^+^) to the rhizosphere or secrete organic acid to maintain pH stability under saline-alkali stress [26,94]. The function of the PM H^+^-ATPase is to establish the H^+^ gradient and thus mediate various secondary active transport events [12,95]. In recent years, H^+^-ATPase (HA) family members have been identified in a wide range of plant species and several advances have been made in the regulation of H^+^-ATPase activity. In Arabidopsis, AHA2 acts as the main pump mediating root H^+^ efflux. Under normal conditions, PKS5 phosphorylates AHA2 at Ser931 to prevent 14-3-3 protein binding with it, thus inhibiting the activity of AHA2. However, under saline-alkali stress, the cytosolic Ca^2+^ increased and the Ca^2+^ binding protein SCaBP1 can interact with PKS5, thus promoting 14-3-3 protein binding with AHA2, releasing auto-inhibition at the C-terminal and ultimately activating AHA2 [20]. The molecular chaperone J3 is also involved in this process, which can inhibit the activity of PKS5, thus activating AHA2 [96]. In addition, alkali stress also triggers the Ca^2+^ signal and induce the Ca^2+^ binding protein SCaBP3 dissociation from AHA2 to promote the activity of PM H^+^-ATPase [26].

The activity of H^+^-ATPase can be regulated both at transcriptional level and post-translational modification in plant response to saline-alkali stress (Figure 2). In apple, the SUMO E3 ligase MdSIZ1 positively regulated the activity of PM H^+^-ATPase and promoted the rhizosphere acidification and Fe uptake, indicating the role of sumoylation in mediating PM H^+^-ATPase activity [97]. In maize, saline-alkali stress induced the expression of *MHA2* and *MHA4* and enhanced the activity of PM H^+^-ATPase, thus promoting root H^+^ efflux and saline-alkali tolerance of maize [58].

The V-H^+^-ATPase and V-H^+^-PPase located in the tonoplast also play a role in mediating the intracellular pH and Na^+^ balance under salt stress. In Arabidopsis, overexpressing *AVP1* that encoded V-H^+^-ATPase enhanced the slat tolerance by increasing the proton gradient across the tonoplast and promoting Na^+^ regionalization into the vacuole [61]. In potato, the activity of V-H^+^-ATPase and V-H^+^-PPase was severely reduced in salt-sensitive cultivars [98]. Therefore, H^+^-ATPase in the plasma membrane or tonoplast coordinates the regulation of pH stability and ion balance under saline-alkali stress in plants (Figure 2).

Organic acid secretion and accumulation is an important strategy adopted by plants to promote the acidification of rhizosphere, thus maintaining pH stability to alleviate the damage to plants caused by high pH stress. In wheat, the accumulated organic acids promote the intracellular ion balance and pH stability under alkali stress [29]. Studies in yellow horn showed that the increased organic acid synthesis gives plants the osmotic tolerance under saline-alkali stress [99]. Metabolic profiling in rice indicated that the transgenic plants (trehalose-6-phosphate synthase/phosphatase, TPSP) accumulated more organic acids and confers plant the high pH (~9.9) tolerance [100]. These studies showed that secretion and accumulation of organic acids at plants rhizosphere plays a critical role in regulating pH stability. To sum up, to cope with the high pH stress induced by saline-alkali, plants activated H^+^-ATPase to increase root H^+^-efflux and accumulated organic acid to acidify the rhizosphere, thus maintaining the pH stability under saline-alkali stress. However, the mechanism still remains largely unknown for the plants that respond to saline-alkali induced high pH stress. It remains elusive that how plants perceive the change of pH and convert it into second messengers (e.g., Ca^2+^), what other key factors regulate PM H^+^-ATPase activity and root acidification through Ca^2+^ signaling, and what are the regulation mechanisms of H^+^-ATPase.

Rhizosphere microorganisms confer fitness advantages to plant abiotic stress tolerance [101]. Saline-alkali stress caused the change of root-soil-microorganism interaction [102], implying that rhizosphere microorganisms can confer plant tolerance to saline-alkali at some extent. In recent years, various species of rhizosphere microorganisms have been demonstrated to improve plant saline-alkali tolerance and crop productivity [103]. Of these, plant growth-promoting rhizobacteria (PGPR) is well-studied compared to other plant-associated microbiota [101]. For example, soil salinization induced high pH stress, which further affects the absorption of nutrients by plants, while *Fluorescent Pseudomonad* reduces the pH of rhizosphere by secreting H^+^ into plants rhizosphere, thereby solubilizing soil minerals. This rhizobacteria can also dissolve phosphorus compounds by releasing organic acids (e.g., gluconic acid, citric acid and succinic acid), thus to alleviate phosphorus deficiency under saline-alkali stress [104,105]. PGPRs were also reported to promote the absorption of Fe in the rhizosphere of plants by producing siderophores (iron chelating agents) to cope with the decrease of Fe solubility induced by high pH value [106]. In addition, the previous study showed that PGPRs (e.g., *Azotobacter* strains) reduce Na^+^ content and increase K^+^/Na^+^ ratio, thus to improve plants salt tolerance [107], suggesting that PGPRs may play an important role in plants response to saline-alkali induced Na^+^ toxicity. PGPRs are involved in accumulating osmolytes, producing ACC deaminase (inhibiting ethylene synthesis) and altering the antioxidant defense system [108,109,110]. Since natural variations in rhizobacteria-plant interaction have been shown to be associated with various agronomic traits [111,112], it would be valuable to explore the association between rhizobacteria-plant interaction and the diversity of plants saline-alkali tolerance, and further elucidate its underlying molecular mechanisms.

## 4. Multiple Signaling Pathways Involved in Response to Saline-Alkali Stress

### 4.1. Plant Hormones Mediate Saline-Alkali Tolerance

**Figure 3:**
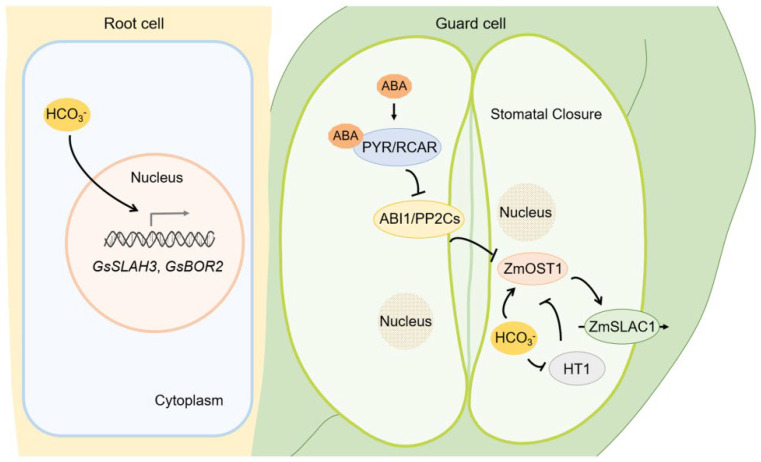
The mechanism model of plant response to HCO_3_^−^/CO_3_^2−^ stress induced by saline-alkali stress. HCO_3_^−^ increased the transcript level of *GsSLAH3* and *GsBOR2* in root, thus promoted the saline-alkali tolerance. Saline-alkali stress induced the accumulation of ABA, which binds the sensor PYR/RCAR and forms a complex with protein PP2C, then release protein kinase OST1 to activate anion channels SLAC1. HCO_3_^−^ also functions as small molecule to active OST1, thus mediates stomatal closure in guard cell and promotes osmotic stress tolerance induced by saline-alkali stress. HT1 protein kinase functions as a negative regulator of high HCO_3_^−^ induced stomatal closing.

Plant hormones are small molecules that play a crucial role in response to abiotic stress (e.g., drought, salt) [113,114]. Saline-alkali stress induce osmotic stress, and ABA functions in plant response to osmotic stress [113,115]. Osmotic stress rapidly induced the endogenous ABA synthesis, which can bind to pyrabactin resistance/pyrabactin resistance-like (PYR/PYL) and forms a complex with protein phosphatase 2C (PP2C), then activating sucrose nonfermenting 1-related protein kinase (SnRK2s). SnRK2 further phosphorylate ABA-responsive element binding protein/ABRE-binding factor (AREB/ABF), regulate ABA-responsive genes expression and promote stomata closure in response to osmotic stress [116,117]. These results suggest that the activation of SnRK2s plays crucial role in ABA-mediated signaling pathway in plants response to osmotic stress. As the upstream components of ABA signaling pathway, B2, B3 and B4 Raf like kinases (RAFs) can be rapidly activated by osmotic stress, and required for the phosphorylation activation of SnRK2s [118,119]. In recent years, great progress has been made in ABA signaling involved in plant response to saline-alkali stress in various plants including Arabidopsis, crops, and woody plants. In Arabidopsis, saline-alkali induced osmotic stress increased ABA accumulation, which binds to PYL and releases protein kinase open stomata (OST1) to activate the slow anion channel-associated (SLAC1) channel and promotes anion efflux and stomatal closure (Figure 3) [120,121,122]. In crops, GWAS were used to identify the genes related to ABA biosynthesis, catabolism, and signaling pathway under saline-alkali stress in *sorghum bicolor*, and saline-alkali treatment increased the expression levels of ABA-related genes (e.g., *SbNCED3*, *SbABAOX1* and *SbPYL7*) [123]. Overexpressing the soybean (*Glycine soja*) SKP1-like family gene *GsSKP21* in Arabidopsis altered the expression levels of ABA signaling-related and ABA-induced genes, decreased ABA sensitivity in seed germination and seedling of early growth stage, and dramatically improved the saline-alkali tolerance of transgenic plants [124]. In woody plants, saline-alkali stress increased the ABA content in apple rootstocks, and the concentration was higher in saline-alkali-tolerant varieties. Transcriptome analysis reveals that the transcript levels of ABA-related genes *CIPK1* and *AHK1* were induced by saline-alkali stress [125]. Studies in *Sophora alopecuroides* showed that the putative genes encoding ABA receptors (*SaPYL4-1*, *SaPYL4-2*, *SaPYL4-3*, *SaPYL4-2* and *SaPYL5-1*) were downregulated after salt and alkali treatments, while the genes encode PP2C (*SaPP2C8* and *SaPP2C53*) were upregulated [126]. These studies demonstrated that ABA acts as the stress-induced hormone involved in the process of plants coping with saline-alkali stress, and the transcription level of ABA synthesis or signaling pathway-related genes was mediated under saline-alkali stress.

H^+^-ATPase functions in acidizing the rhizosphere to maintain pH stability under saline-alkali stress. It was reported that auxin (IAA) can regulate PM H^+^-ATPase activity. In Arabidopsis, alkali stress (pH 8.0) increased PIN2 abundance in the root tip and promoted auxin transport, while the activity of PM H^+^-ATPase decreased in *pin2* and *pin2 pks5* [127]. Overexpressing *GsERF71* in Arabidopsis promoted auxin accumulation and induced the expression of H^+^-APTase, thus significantly enhancing the saline-alkali tolerance of transgenic plants [128]. Studies in apples also showed that under saline-alkali stress, IAA concentrations and transcript levels of IAA-related genes *ARF5*, *GH3.6*, *SAUR36* were significantly higher in saline-alkali tolerant varieties [125]. These results suggest that auxin promote plants saline-alkali tolerance by involved in regulating PM H^+^-ATPase activity. In addition, a recent study revealed that IAA plays a role in saline-alkali tolerance by modulating root development and ROS detoxifying systems in Rice [123].

Ethylene (Eth) was the first identified gaseous plant hormone, which has been reported as a positive regulator for salt and saline-alkali tolerance [129]. However, a recent study in rice showed that during the period of post-anthesis, saline-alkali stress induces the biosynthesis of Eth, which has a negative effect on grain filling [130], suggesting a diverse roles in plant response to saline-alkali stress during different development stages. Studies in Arabidopsis showed that under saline-alkali stress, Eth increased the transcript level of *AUX1* and auxin biosynthesis-related genes, promoted auxin accumulation, and relieved the inhibition of root elongation under saline-alkali stress [131]. These results suggest that Eth and IAA synergistically regulate plant saline-alkali tolerance.

In addition, gibberellin (GA), jasmonic acid (JA), salicylic acid (SA) and brassinosteroid (BR) have also been reported to be involved in plants responding to saline-alkali stress. In rice, the concentration of endogenous gibberellin (GA) and the transcript levels of GA biosynthetic genes were greatly higher in tolerant varieties under saline-alkali stress. The accumulation of endogenous GAs confers the saline-alkali tolerance during seed germination in rice [132]. Overexpression of a JASMONATE ZIM-DOMAIN (JAZ) family protein, GsJAZ2, increases the saline-alkali tolerance in transgenic Arabidopsis plants, suggesting that jasmonate may play a role in plant response to saline-alkali stress [133]. Exogenous application of JA, SA, and BR alleviate the damage caused by salt and alkali stress in plants by alleviating growth inhibition, ion toxicity or maintaining ROS homeostasis, etc. [134,135]. Saline-alkali stress induced multiple hormone-related genes expressions. For example, *AUX/IAA*, *A-ARR*, *ABF*, *PP2C*, *EIN3*, *JAZ* or *TGA* was strongly induced by NaHCO_3_/Na_2_CO_3_ treatment in wheat [136]. In sorghum, saline-alkali stress significantly increased the expression level of ABA signaling related genes, including *SbNCED3*, *SbPP2C09*, *SbPP2C23*, *SbPP2C52*, *SbPP2C54*, *SbPP2C58*, *SbSAPK1*, *SbSAPK5*, and *SbSAPK9* [123]. These results indicated that multiple plant hormones signaling synergistically mediate plant response to saline-alkali stress. However, the hormone-mediated gene expression network and their crosstalk between saline-alkali signaling is still largely unknown, which needs further investigation.

### 4.2. ROS Signaling and Antioxidants

Plants grown under saline-alkali conditions also suffer from oxidative stress, which causes metabolic disorder and disrupts the normal physiological functions of plants [137]. Oxidative damage was mainly caused by reactive oxygen species (ROS) excessive accumulation. In order to cope with the oxidative damage, plants usually activate ROS scavenging systems that include antioxidant enzymes and antioxidants, which play a role in maintaining ROS homeostasis [1,3].

A large number of studies showed that increasing the activity of antioxidant enzymes and increasing the content of antioxidants prevent excessive accumulation of ROS and alleviate oxidative damage caused by saline-alkali stress. Overexpressing the sulfite reductase gene *MsSiR* promote the synthesis of glutathione reduced (GSH) and positively regulate the detoxification and antioxidant process, thus enhancing the resistance to saline-alkali stress [138]. Similarly, overexpressing *GmPKS4* in Arabidopsis activate ROS scavenging systems and improved the saline-alkali tolerance [139]. The transfer of the type 2 metallothionein gene *SsMT2* from *Suaeda salsa* into Arabidopsis activates ROS scavenging pathway and reduces the content of H_2_O_2_, ultimately improving plant oxidant tolerance caused by saline-alkali stress [140]. Some transcription factors were also identified to participate in the regulation of ROS scavenging. For instance, ectopic expression of *SlWRKY28* from *Salix linearistipularis* into *Populus davidiana* × *P. bolleana* induced the expression of ROS scavenging pathway-related genes, thus enhancing the tolerance of transgenic plants to saline-alkalis stress [141]. In the future, more transcriptional factors in this field should be identified for molecular breeding to increase plant tolerance to saline-alkali stress.

## 5. HCO_3_^−^/CO_3_^2−^ Stress Caused by Saline-Alkali Stress

Basic salts are mainly composed of NaHCO_3_ and Na_2_CO_3_, which contributed to the increase the pH value in soil. However, a body of evidence suggests that plant responding to HCO_3_^−^/CO_3_^2−^ stress is not exactly the same as high pH stress. Up to now, some transporters have been identified to participate in HCO_3_^−^/CO_3_^2−^ stress. Boron (B) is an essential microelement in higher plants, and B deficiency increased K^+^ leakage and decreased nitrate uptake, thus affecting plants growth and crop yields [142,143,144]. High boron requiring (BOR) transporters have been reported to be involved in response to saline-alkali stress in *Glycine soja*. The expression of *GsBOR2* can be induced by NaHCO_3_ treatment and overexpressing *GsBOR2* in Arabidopsis enhanced the resistance to NaHCO_3_ and KHCO_3_ treatment [145]. The transgenic Arabidopsis overexpressing a slow-type anion channel GsSLAH3 also confers the tolerance to HCO_3_^−^ stress [146]. Interestingly, overexpression of *GsBOR2* or GsSLAH3 in Arabidopsis showed no resistance to high pH stress. These studies suggest that BOR2 and SLAH3 positively regulates saline-alkali stress and specifically enhances HCO_3_^−^ resistance. Furthermore, some transcription factors such as GsERF6, GsERF71, GsbZIP67 and HD-Zip *Gshdz4* were identified to increase specific HCO_3_^−^ tolerance under saline-alkali stress [128,147,148,149]. *GsbZIP67* was identified from *Glycine soja* as a HCO_3_^−^-responsive gene, overexpressing of *GsbZIP67* in alfalfa promotes plant growth under saline-alkali stress, suggesting that GsbZIP67 positively regulates saline-alkali tolerance by enhancing plants tolerance to HCO_3_^−^ [149]. The Ca^2+^ signaling pathway was also involved in plant response to HCO_3_^−^ stress, probably independent of salt stress and osmotic stress. In soybean, *GsCML27* encodes a Calmodulin-like family proteins (CMLs) and its expression can be induced by HCO_3_^−^, salt and osmotic stresses, while ectopic expression of *GsCML27* in Arabidopsis increased plants’ HCO_3_^−^ tolerance, but decreased the salt and osmotic tolerance [150]. In addition, HCO_3_^−^ also acts as a small molecule to activate SLAC1 anion channels and mediates stomatal closure in guard cells, suggesting that HCO_3_^−^ probably participates in the response to osmotic stress induced by saline-alkali [151,152] (Figure 3).

Above all, existing studies have shown that plants adapt to HCO_3_^−^/CO_3_^2−^ stress not exactly the same as high pH stress. Compared to high pH values, plants seemed to adopt more complex mechanisms to cope with HCO_3_^−^/CO_3_^2−^ stress. Our recent study also found an interesting phenomenon in blueberry, a variety with high pH tolerance showed resistance to basic salt (NaHCO_3_) but hypersensitive to neutral salt (NaCl or Na_2_SO_4_), while another pH-sensitive variety showed opposite (data unpublished). Therefore, we conjectured that there may be an interaction between HCO_3_^−^/CO_3_^2−^ and high pH, or HCO_3_^−^ probably acts as a signaling molecule, and then jointly promote Na^+^ shoot exclusion in plants. However, in contrast to the relatively in-depth understanding of plant in response to salt or high pH stress, little is known about how plant responding to HCO_3_^−^/CO_3_^2−^ stress. Using pH or salt-tolerant or sensitive plant species or varieties, and exploring the mechanisms of differential response to combined saline-alkali stress would be helpful in the future to uncover the mystery.

## 6. Other Understandings of Plant Saline-Alkaline Tolerance

Saline-alkaline soil shows a severe lack of nutrition and the sufficient supply of mineral nutrition to some extent alleviates the symptoms in plants caused by saline-alkali stress. Studies in saline-alkali tolerant plants *Puccinellia tenuiflora* showed that the genes involved in mineral uptake were significantly increased and exogenous application of nutrition elements increased the saline-alkali tolerance of rice, suggesting that nutrition uptake can enhance plants’ saline-alkali tolerance [153]. Among the multiple nutrients, efficient supply of Fe is likely to be related to plant saline-alkali tolerance. For example, in rice, Fe concentration was higher in the saline-alkali tolerant variety, and Fe-deficiency-responsive genes *OsIRO2*, *OsIRT1*, *OsNAS1*, *OsNAS2*, *OsYSL2*, and *OsYSL15* were significantly upregulated under saline-alkali stress [154]. RNA-seq analysis in *Puccinellia tenuiflora* also showed that saline-alkali stress induced the expression of genes involved in Fe acquisition [155], suggesting that highly efficient Fe acquisition provides plants with a greater extent of HCO_3_^−^ tolerance, and maintaining sufficient soluble Fe may well be one of a key factor in improving plants saline-alkali tolerance.

## 7. Saline-Alkali-Tolerant Breeding

**Figure 4:**
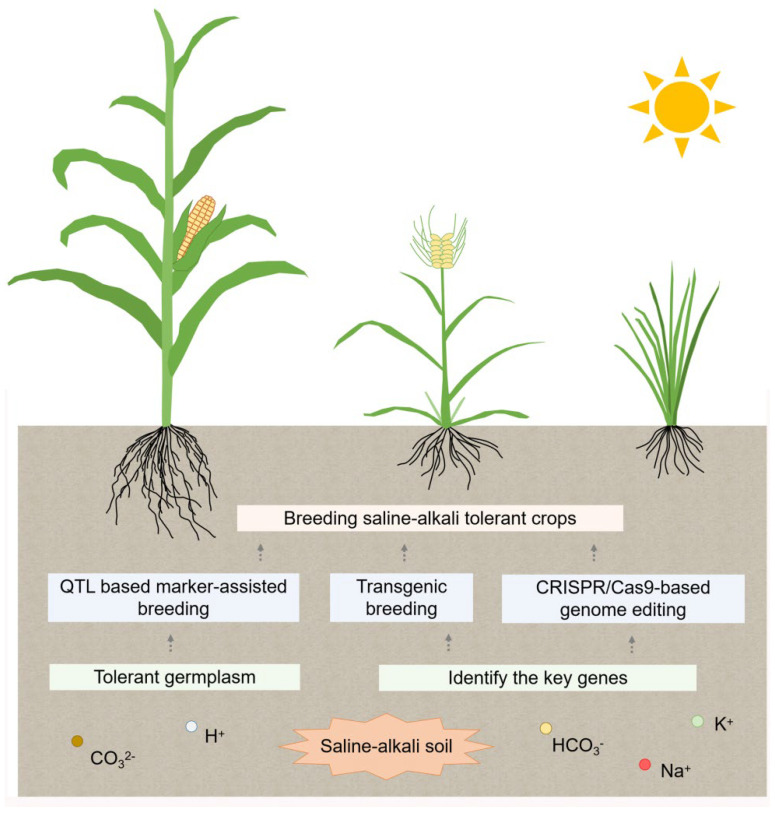
Schematic diagram of developing saline-alkali tolerant crops based on modern breeding. Screening the saline-alkali tolerant germplasm, and identifying key determinants that regulate plants saline-alkali tolerance based on QTL, GWAS analysis, etc. Then introgression/transfer favorable alleles into elite varieties to develop saline-alkali tolerant crops by modern breeding approach, which include QTL based marker-assisted breeding (MAS), transgenic breeding and CRISPR/Cas9-based genome editing technique.

According to the statistics, nearly 50% of the farmland would suffer from soil salinization by 2050, which will become a global issue threatening crops (e.g., rice, wheat and maize) productivity [156]. Developing saline-alkali-tolerant crops is the most fundamental and efficient strategy to cope with soil salinization and guarantee food security. Beginning in the early part of this century, breeders attempted to improve the crops saline-alkaline tolerance through conventional breeding, whereas received limited success owing to the complexity of plants’ response to saline-alkali stress [157]. In the recent decades, the rapid development of molecular genetics and functional genomics have made it possible to excavation favorable alleles for saline-alkali tolerance and to introgress/transfer them into elite varieties [137,157]. The main approaches include QTL based marker-assisted breeding (MAS), transgenic breeding and CRISPR/Cas9-based genome editing technique (Figure 4). Meanwhile, the advance of saline-alkali-response mechanisms in plants (Figure 2 and Figure 3) forms the basis for developing effective approaches to improve crops saline-alkali tolerance. Based on this, some progress has also been made in breeding commercial varieties of saline-alkali-tolerant crops [157,158].

### 7.1. Transgenic Breeding

Transgenic approach has been proved as a potential way of achieving saline-alkali tolerance in crops. For the key determinants of plants response to saline-alkaline stress, overexpression of a single key gene can improve plants saline-alkali tolerance to a certain extent. For example, transgenic plants overexpressing *OsSDG721* showed more tolerant under saline-alkali stress, which attribute to OsSDG721 positively regulating the expression level of OsHKT1;5, thus maintaining K^+^/Na^+^ homeostasis [79]. Ectopic expression of *AVP1* (Arabidopsis vacuolar H^+^-pyrophosphatase) in barley increased plants shoot biomass and grain yield in the saline field [159]. In maize, overexpression of *ZmCS3* (citrate synthase) and *ZmUGT* (glucosyltransferase) promote the accumulation of metabolites and salt-induced osmotic stress tolerance [16]. Although some genes that play an essential role in plant response to salt or saline-alkaline stress, the lack of these genes leads to plants salt-hypersensitive, overexpression of them only slightly improves salt tolerance, such as SOS pathway genes and salt-induced osmotic stress-related gene *ZmCYP709B2* (cytochrome P450 polypeptide) [16,19,160]. Therefore, it is very essential to identify the key determinants that can regulate the optimal spatiotemporal expression of salt-tolerant genes, and transform multiple genes to improve crops salt tolerance in the long term.

### 7.2. QTL Based Marker-Assisted Breeding

Plant saline-alkali tolerance is a complex trait controlled by multiple quantitative genes (QTLs) and mutation or overexpression of a single gene rarely significantly improve plant salt-alkali tolerance [19,160,161]. Marker-assisted selection (MAS) and marker assisted backcrossing (MABC) breeding can conveniently transfer major saline-alkali-tolerant loci into elite varieties [157,162]. Modern breeding combined QTL analyses with marker-assisted selection accelerate and more accurately identify linked genes for saline-alkali tolerance, which has been successfully applied for saline-alkali-tolerant crops breeding [157,158,162,163,164]. For example, *Saltol* locus is responsible for rice shoot Na^+^/K^+^ homeostasis under salt stress, in this region contains a QTL gene *SKC1* that encodes a Na^+^-selective transporter OsHKT1;5, which has been successfully introgressed into commercial varieties through marker-assisted breeding [74,164,165]. *Kna1* locus in bread wheat has been identified as *TaHKT1;5-D*, *Nax1* and *Nax2* locus in durum wheat were identified as TmHKT1;4 and TmHKT1;5-A, respectively. These genes encode Class I HKT family protein, and function in regulating Na^+^/K^+^ homeostasis and plant salt tolerance [75,166,167,168,169,170]. Introgression of *Nax2* into commercial durum wheat significantly reduced leaf Na^+^ content and increased grain yield by 25% in saline soils [158]. The rapid development of next-generation sequencing (NGS) facilitates the precise location and quicker cloning of QTLs for crop saline-alkali tolerance. In recent years, numerous QTL genes contributed to salt or saline-alkali tolerance were also identified in maize, including *ZmNC1*, *ZmNC2*, *ZmNC3* and *ZmSOS1* that regulate Na^+^ homeostasis, *qKC3* functioning in maintaining K^+^/Na^+^ homeostasis, *ZmNSA1* involved in shoot Na^+^ exclusion under saline-alkali stress, and other several genes (e.g., *ZmCS3*, *ZmUGT* and *ZmCYP709B2*) conferring to the accumulation of osmoregulatory substances, which provide gene resources for breeding salt-tolerant maize varieties [16,47,58,72,73,89,90]. This outcome has proved that QTL based marker-assisted breeding is effective for developing saline-alkali-tolerant crops.

### 7.3. CRISPR/Cas9-Based Genome Editing Technology

Despite the effective application of transgenic breeding and QTL based marker-assisted breeding for salt-tolerant crops, the superiority is still limited. Genes with specific spatiotemporal expression play a crucial role in transgenic-based breeding, such as HKT1, whose expression only in stele can improve salinity tolerance, while marker-assisted-selection based breeding has probably caused undesirable traits to be transferred together with QTL locus and affect agronomic traits [137,171]. Therefore, it is essential how precisely manipulate these genes to function in target crops. Genome editing technology is an emerging and revolutionary approach for accelerating crop breeding. With the rapid development of functional genomics, CRISPR/Cas9 technology including knockout, base editing and allele exchange has been effectively utilized in improving major crops salt tolerance. For example, knocking out *OsRR22* (B-type cytokinin transcription factor) significantly enhanced rice salt tolerance whereas not affected agronomic traits [172]. In maize, knocking out K^+^-preferring ZmHKT2 (Class II HKT family transporter) enhanced shoot K^+^/Na^+^ ratio, and promoted maize salt tolerance [73]. The mutants of ZmCYP709B2 (cytochrome P450 polypeptide) maintained greater leaf water contents and were more tolerant to salt stress [16]. In addition, using multiplex CRISPR-Cas9 editing system can modify specific regions (e.g., coding sequences, cis regulatory regions, upstream open reading frames) of several genes simultaneously. Genome edited tomato with four genes (*SP*, *SP5G*, *SlCLV3* and *SlWUS*) not only exhibited domesticated phenotypes but retained wild parental salt tolerance [173]. Previous studies showed that numerous key genes negatively regulate plant saline-alkali tolerance. For example, ZmNSA1 negatively regulates the activity of PM H^+^-ATPase and SOS1, functions as a negative regulator of maize saline-alkali tolerance [58]. In rice, the ethylene biosynthesis-related genes, *OsACS* and *OsACO*, also play negative roles in response to saline-alkali stress during the period of post-anthesis [130]. PsnWRKY70 negatively regulate saline-alkali tolerance by affecting the cell wall organization or biogenesis-related gene expression in Poplar [174]. Therefore, the CRISPR/Cas9-mediated multiplex genome editing system provides a more powerful and efficient tool to generate saline-alkali-tolerant crops using these genes as target genes.

Although several QTLs have been successfully applied for breeding salt-tolerant crops, more specific saline-alkali QTLs still need to be explored. Notably, plants that respond to saline-alkali stress are usually regulated by a combination of factors, and the growth conditions are very different from that under the real field conditions (e.g., humidity, soil composition, etc.). Therefore, in addition to focusing on the molecular mechanisms of saline-alkali tolerance, the gap between greenhouse and field cultivation is still not filled up and should be considered in the future research.

## 8. Concluding Remarks and Future Prospects

To sum up, great progress has been made in unveiling the molecular mechanism of plant response to salt stress, but little attention has been paid to saline-alkali combined stress. We listed the genes that are involved in plants responding to saline-alkali stress and summarized the progress in understanding the plant saline-alkali tolerance. However, there are still some issues that require broader efforts in future research.

(1) How plant perceive the saline-alkali stress?

The putative sensor of osmotic stress (OSCA1), Na^+^ (GIPC) and extracellular pH (RGF1-RGFRs and Pep1-PEPRs) have been identified in previous studies, whereas it remains largely unknown how plants sense HCO_3_^−^/CO_3_^2−^ stress. In the future, the efforts should be made to identify the sensors of saline-alkali stress, and clarify how plants sense the change in HCO_3_^−^/CO_3_^2−^.

(2) Identification of Na^+^ transporters that specifically function under saline-alkali stress.

Plants grown under saline-alkali conditions accumulated excessive Na^+^ and led to ion toxicity. Na^+^-preferring transporters enable the circumvention of Na^+^ toxicity (Figure 2). Plants accumulated more Na^+^ under saline-alkali stress than salt stress [58]. Therefore, whether certain Na^+^ transporters play a specific role under salt or saline-alkali stress, whether the identified key Na^+^ transporters showed distinct activity under different treatments remains to be elucidated.

(3) Determine if there are common key regulators under high pH and HCO_3_^−^/CO_3_^2−^ stress.

The high pH of salinization soil was mainly caused by basic salt (e.g., NaHCO_3_, Na_2_CO_3_). Recent studies suggest that plants probably sense the change in pH and HCO_3_^−^/CO_3_^2−^ through different pathways, but whether the common key regulators exist and the specific response mechanism still need to be further studied.

(4) How to use the molecular markers to cultivate saline-alkali resistant crop varieties?

Plant saline-alkali tolerance is a complex trait controlled by multiple quantitative genes (QTLs) and mutation or overexpression of a gene does not significantly improve plant saline-alkali tolerance. Therefore, GWAS and QTL analysis should be used to identify new determinant genes and molecular makers to promote saline-alkali tolerant breeding. In addition, crop yield should be guaranteed when the plants’ saline-alkali tolerance is improved. Therefore, whether the molecular makers and QTLs influence the other agronomic traits should also be considered in the future breeding objectives.

In conclusion, future research should focus on dissecting the mechanisms of saline-alkali combined stress, including that how plants perceive saline-alkali stress and how they maintain Na^+^ homeostasis under saline-alkali stress. More importantly, by elucidating the interaction between pH and HCO_3_^−^/CO_3_^2−^ stress, the superior alleles of saline-alkali stress would be excavated and used to cultivate saline-alkali-tolerant crops.

## Figures and Tables

**Table 1:** List of genes likely associated with saline-alkaline (NaHCO_3_ and Na_2_CO_3_) tolerance in plants.

Gene Name	Plant Species	Type of Saline-Alkaline Stress	Response Stress	Genetic Evidence	Reference
*OsSDG721*	*Rice* (*Oryza sativa*)	Na_2_CO_3_	Na^+^ toxicity	T-DNA insertion and overexpression in rice	Liu et al., 2021
*OsHKT1;5*	*Rice* (*Oryza sativa*)	Na_2_CO_3_ or NaCl	Na^+^ toxicity	Crispr/Cas9 technology-generated mutant in rice	Kobayashi et al., 2017; Chuamnakthong et al., 2019; Liu et al., 2021
*OsDMI3*	*Rice* (*Oryza sativa*)	NaHCO_3_	Na^+^ toxicity	Knock out and overexpression in rice	Ni et al., 2021
*GmMYB3a*	Soybean (*Glycine max*)	NaCl or Na_2_CO_3_	Osmotic stress	overexpression in Soybean	He et al., 2018
*GsSKP21*	Soybean (*Glycine soja*)	NaHCO_3_	Osmotic stress	overexpression in Arabidopsis	Liu et al., 2015
*CrPIP2;3*	*Canavalia rosea*	NaHCO_3_	Osmotic stress	overexpression in Arabidopsis	Zheng et al., 2021
*VaERF3*	adzuki bean	NaHCO_3_	Osmotic stress oxidative damage	overexpression in Arabidopsis	Li, et al., 2020
*ZmNSA1*	maize	NaHCO_3_	Na^+^ toxicity	UF-Mu insertion and overexpression in maize	Cao et al., 2020
*LcCHI2*	*Leymus chinensis*	Na_2_CO_3_	Na^+^ toxicity	overexpression in tobacco and maize	Liu et al., 2020
*GsPPCK3*	Glycine soja	NaHCO_3_	high pH stress	overexpression in alfalfa	Sun et al., 2014
PKS5	Arabidopsis	High pH	high pH stress	T-DNA insertion mutant in Arabidopsis	Fuglsang et al., 2007
J3	Arabidopsis	NaCl combined with high pH	high pH stress	T-DNA insertion mutant in Arabidopsis	Yang et al., 2010
*GsJAZ2*	Soybean (*Glycine soja*)	NaCl or NaHCO_3_	-	overexpression in Arabidopsis	Zhu et al., 2012
*GsERF6*	Soybean (*Glycine soja*)	NaHCO_3_ or KHCO_3_	bicarbonate stress	overexpression in Arabidopsis	Yu et al., 2016
*GsERF71*	Soybean (*Glycine soja*)	NaHCO_3_ or KHCO_3_	bicarbonate stress	overexpression in Arabidopsis	Yu et al., 2017
*Gshdz4*	Soybean (*Glycine soja*)	NaHCO_3_ or KHCO_3_	bicarbonate stress	overexpression in Arabidopsis	Cao et al., 2016
*GsBOR2*	Soybean (*Glycine soja*)	NaHCO_3_ or KHCO_3_	bicarbonate stress	overexpression in Arabidopsis	Duan et al., 2018a
*GsSLAH3*	Soybean (*Glycine soja*)	NaHCO_3_ or KHCO_3_	bicarbonate stress	overexpression in Arabidopsis	Duan et al., 2018b
*GsCML27*	Soybean (*Glycine soja*)	NaHCO_3_	bicarbonate stress	overexpression in Arabidopsis	Chen et al., 2015
*GsbZIP67*	Soybean (*Glycine soja*)	NaHCO_3_	bicarbonate stress	overexpression in *alfalfa*	Wu et al., 2018
*SlWRKY28*	*Salix linearistipularis*	NaHCO_3_	oxidative damage	overexpression in *Populus davidiana* × *P. bolleana*	Wang et al., 2020b
*GmPKS4*	Soybean (*Glycine max*)	NaCl or NaHCO_3_	oxidative damage	overexpression in Soybean and Arabidopsis	Ketehouli et al., 2021
*GsNAC019*	Soybean (*Glycine soja*)	NaHCO_3_	-	overexpression in Arabidopsis	Cao et al., 2017
*SsMT2*	*Suaeda salsa*	NaHCO_3_	oxidative damage	overexpression in Arabidopsis	Jin et al., 2017
